# Genetic and structural characterization of the growth hormone gene and protein from tench, *Tinca tinca*

**DOI:** 10.1007/s10695-012-9661-x

**Published:** 2012-06-02

**Authors:** R. Panicz, J. Sadowski, R. Drozd

**Affiliations:** 1 Department of Aquaculture, West Pomeranian University of Technology, Szczecin, Kazimierza Krolewicza 4, 71-550 Szczecin, Poland; 2 Department of Immunology, Microbiology and Physiological Chemistry, West Pomeranian University of Technology, Szczecin, Doktora Judyma 24, 71-466 Szczecin, Poland

**Keywords:** Growth hormone, Growth rate, Protein structure modelling, Species diversity, Tench

## Abstract

The analysis of the tench growth hormone gene structure revealed a comparable organization of coding and non-coding regions than other from cyprinid species. Based on the performed mRNA and amino acid sequence alignments, *gh* tench is related to Asian than to European representatives of Cyprinidae family. Second aim of the work was to characterize and predict protein structure of the tench growth hormone. *Tinca tinca GH* share many common features with human GH molecule. The Tench GH protein binds to the growth hormone receptor (GHR) using two regions I and II that are situated at opposite sites of molecule. Binding site I is placed in the central part of *T. tinca* GH and H 189 amino acid in the middle region of the IV helix is crucial for GH–GHR interactions.

## Introduction

The Tench, *Tinca tinca* L., is a warm-water cyprinid fish commonly found in Europe and adjacent regions , although human activities are wide spread. Outside of Europe, the tench is found in the continents of North America (USA, Canada), South America (Chile), Africa (Tunisia, South Africa, Zimbabwe, Zambia) and Australia (Tasmania) as well as in Japan, Indonesia and New Zealand (Rowe 2004). The Tench generally prefers lower parts of stagnant waters (turbid, lentic) where they live on the muddy ground. During daytime, they tend to hide between plants, roots or other shelters. The Tench is a typical omnivorous species that is looking for food during the night-time using rather olfactory than visual cues (Herrero et al. 2003; Owen et al. 2010). Since centuries, the tench has been cultivated in polish fish farms mainly in polycultures with common carp (*Cyprinus carpio*) to improve its slow growth rate (Skrzypczak and Mamcarz 2006). Except one example, the Quolsdorf tench line this fish has always been considered as a non-profitable species, which needs much more time and care to gain an appropriate weight than other fish. The Quolsdorf tench was a fast-growing strain in early ages of XX century, which was established throughout long-term selection programme. However, after a few years of farming, this strain disappeared and never has been re-established despite huge demand for its meat (Milkau 1921; Müller 1961; Šestáková et al. 1989; Billard and Flajshans 1995). Since that time, many researchers have tried to find a solution to increase the low growth rate of tench and make its culture profitable. The idea of compensatory growth was tested as a possible way to achieve better results via feeding trials for juvenile stages. The initially obtained results were promising; however, it turned out that it was an insufficient attempt to solve this complex problem (Quirós and Alvariño 2000; Wolnicki et al. 2003, 2006; Celada et al. 2008). An approach experimenting with chromosomal manipulations, carried out by Flajshans et al. (1993, 2004, 2007), increased the specific growth rate of tench. However, according to authors’ conclusions, additional analysis of alloenzymes and nuclear DNA must be performed.

The slow growth rate is affected by many factors and should be analysed using various methods. Nowadays, scientists use nutrigenomics to approach such problems because this is a multidisciplinary platform which analyse influence of nutrients on gene expression level (Leaver et al. 2008). In order to analyse and improve the slow tench growth, the most important factors in the pathway that is determining the somatotropic axis. Signals from the hypothalamus are carried to specific tissue receptors responsible for growth, osmoregulation, steroidogenesis or gametogenesis (Mommsen 2001; Sakamoto and McCormick 2006; Canosa et al. 2007). Growth hormone (GH) is regarded as a main component of this axis; however, insulin-like growth factors type I and type II, somatostatin, somatoliberin or specific receptors are equally important. Piscine components of somatotropic axis have been extensively studied and described by Reinecke et al. (2005), and the intricate interactions along this axis were characterized by Wood et al. (2005). A first detailed information for the tench growth hormone gene has been presented by Kocour and Kohlmann (2011). However, in order to study the specific molecular factors that might modulate interactions between hormones and their receptors, it is necessary to gain knowledge about three-dimensional structure (3D).

Considering the facts mentioned above, this study aims to analyse the tench growth hormone gene sequence and to predict the currently unknown, 3D structure of GH protein. Additionally, to define which amino acids in protein structure might be important for internalization of the GH-GHR complex. Information on the 3D structure will be used to emphasize to which extent the results help to improve the knowledge of aquaculture.

## Materials and methods

### Tench *gh* sequence determination

Tench (*T. tinca*) samples, by means of electrofishing, were collected during June of 2008 from Południewo Lake, part of the Masurian Lake District in NE, Poland. Południewo Lake has been chosen because this lake has never been subject to any fishery activities (fishing, fish introductions, annual fish catch, etc.). A total of 29 tench individuals were caught alive, and a small caudal fin-clip dissected from each fish before the fish was released. Total DNA from these samples was extracted using peqGOLD Tissue DNA Kit (PEQLAB Biotechnologie). Purity and concentration of DNA extracts were analysed on a 1 % agarose gel and Nanodrop ND-1000 (Thermo Fisher Scientific Inc.) spectrophotometer, then stored in −20 °C until PCR assays.

**Fig. 1:**
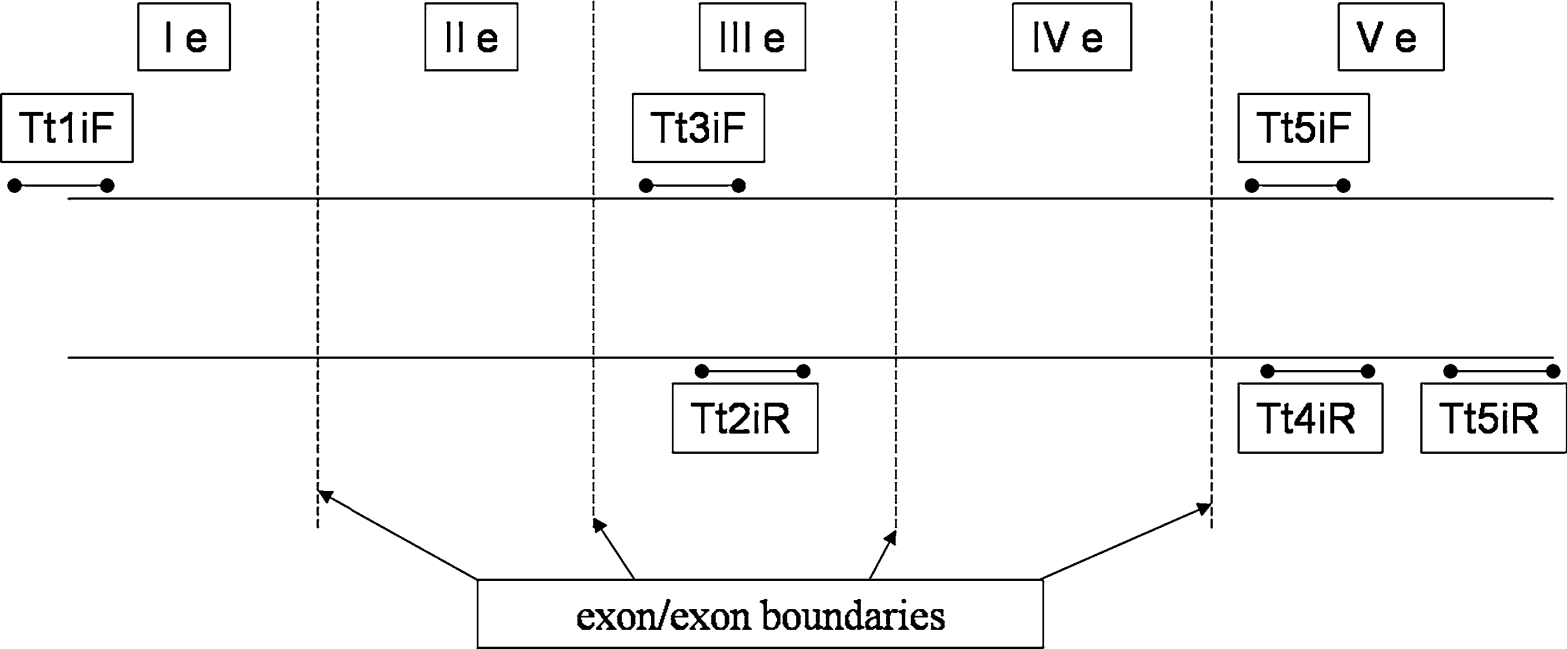
Primer hybridization sites along the *gh* mRNA of the tench. Ie–Ve—particular exons

Primers were designed based on alignment of tench mRNA *gh* (NCBI Acc. Nr. DQ980027) and silver carp (*Hypophthalmichthys molitrix*) growth hormone gene (NCBI Acc. Nr. M94348) sequences. This step determined exon–exon boundaries (vertical dash lines) within tench mRNA sequence, and Primer 3 software generated 3 pairs of primers (Rozen and Skaletsky 2000). Figure 1 illustrates primer hybridization sites.

PCR cycling conditions were as follows: 94 °C/3 min followed by 35 cycles of 94 °C/30 s, depending on primer pair (I—60 °C, II—65 °C and III—57 °C) throughout 30 s, 72 °C/1 min and one cycle of final elongation 72 °C/7 min. Annealing temperatures proposed for each primer pair by Primer 3 software were verified and determined using gradient PCR.

Each PCR product without any additional bands was purified with peqGOLD Gel Extraction Kit (PEQLAB) and bidirectionally sequenced in a CEQ 8000 sequencer (Beckman Coulter). Conditions and reaction mixtures were done according to GenomeLab DTCS Quick Start Kit (Beckman Coulter). Subsequently, mRNA *gh* sequences of tench and other fish from Cyprinidae family were aligned to asses interspecies genetic similarities. DNA sequences were analysed by means of CEQ 8000 Sequence Analysis Software Package, BioEdit and MEGA5 software (Hall 1999; Tamura et al. 2011).

### Tench GH secondary structure prediction and fold recognition

After sequencing, the PCR product was translated to amino acid sequence in BioEdit software. Sequence was used as target for protein structure prediction and homologue searching in databases. Homologue searching was performed in the NR (non-redundant) database set by Psi-BLAST, 5 iteration and e value—0.005 (Altschul et al. 1997). For secondary structure prediction and fold recognition, full-length amino acid sequence was submitted to Meta-server Bioinfobank (Ginalski et al. 2003). Secondary structure was predicted by Psi-Pred (Jones 1999). Available profile–profile servers were used for structural homologue’s searching: FFAS03 (Jaroszewski et al. 2005) and GRDB (Ginalski et al. 2003), and the threading servers FUGUE (Shi et al. 2001) and Sam-T02 (Karplus 2009).

### Homology modelling

Alignments from the fold recognition step with significance scores between target sequence and recognized templates were used for 3D structure modelling. Alignments were optimized manually in the Swiss-PdbViewer 4.0 (Guex and Peitsch 1997), with priority on reducing misalignments and gaps in the protein core regions. Finally, preliminary models were built using MODELLER 9v8 (Eswar et al. 2006). Models were evaluated by the ProQ and the MetaMQAPII server. For further analysis, 3D structures with the best overall score were chosen (Björn and Elofsson 2003; Pawlowski et al. 2008).

### Model analysis

The ConSurf server was used to map conserved regions on molecular surface a modelled GH (Ashkenazy et al. 2010). In order to calculate electrostatic potential on molecular surface, a model of the GH was submitted to PROPKA server to predict the protonation state at pH 7.0 (Li et al. 2005). The Adaptive Poisson–Boltzmann Solver (APBS) software was used for the electrostatic potential map calculation via PROPKA server (Baker et al. 2001). Structures and analysis were visualized using UCSF-Chimera 1.5 (Pettersen et al. 2004).

## Results

### The tench growth hormone gene

The electrophoretic separation of 29 DNA isolates revealed a high purity and concentration of the samples; this was confirmed with the Nanodrop ND-1000. The purity of the extracted nucleic acids has been assessed on the basis of OD 260/280 ratio and ranged from 1.8 to 2.0 for all the samples. PCR and further bidirectional sequencing reactions yielded 174 high-quality reads, which after assembly gave 29 sequences representing the complete growth hormone gene sequence for each individual. Multiple alignment using ClustalW (via BioEdit software) suggested that the 29 growth hormone gene sequences were identical.

**Fig. 2:**
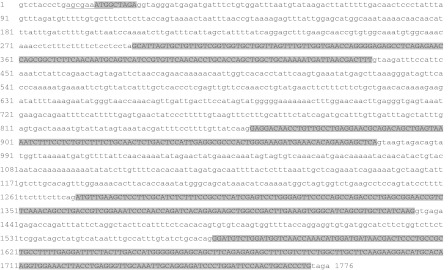
Nucleotide sequence of tench growth hormone sequence determined in this study (description in text)

The nucleotide sequence of tench growth hormone gene that was determined in this study (1,776 bp) has been submitted in GenBank under accession number HM114351.1. The coding region of the sequence consists of four introns indicated by low letters (269, 424, 296 and 137 bp) and five exons shown by shaded capital letters (9, 141, 117, 162 and 201 bp). The open reading frame has 630 nucleotides, including a typical Kozak site (underlined sequence region) and ends with the TAG stop codon (Fig. 2).

**Fig. 3:**
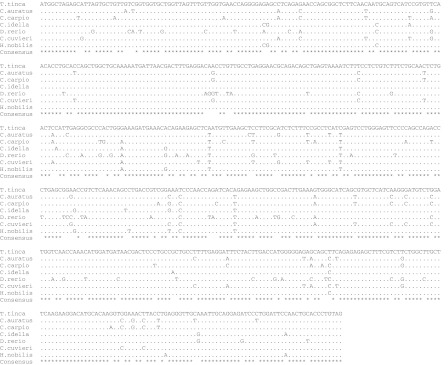
Alignment of seven *gh* mRNA sequences from members of the Cyprinidae family (*Carassius auratus*—AY265352.1, *C. carpio*—M27000.1, *Ctenopharyngodon idella*—AY616661.1, *Danio rerio*—AJ937858.1, *Carassius cuvieri*—AF389237.1, *H. molitrix*—EU157194.1 and *Hypophthalmichthys nobilis*—X60473.1)

**Fig. 4:**
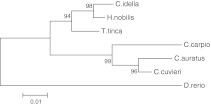
Phylogenetic tree prepared based on the Jukes-Cantor model shows the tench relationship to other species from the *Cyprinidae* family

Based on the alignment of the tench *gh,* mRNA sequence with six others species representing the Cyprinidae family (Fig. 3). The tench shared the highest similarity to bighead carp and grass carp. The similarity to those two species based on the nucleotide level was 97.6 and 97.1 %, respectively (Fig. 4). These results clearly suggest that the tench is more closely related to species that were imported from China and introduced into polish aquaculture than to native Polish (European) species.

### Tench GH protein homology modelling

**Fig. 5:**
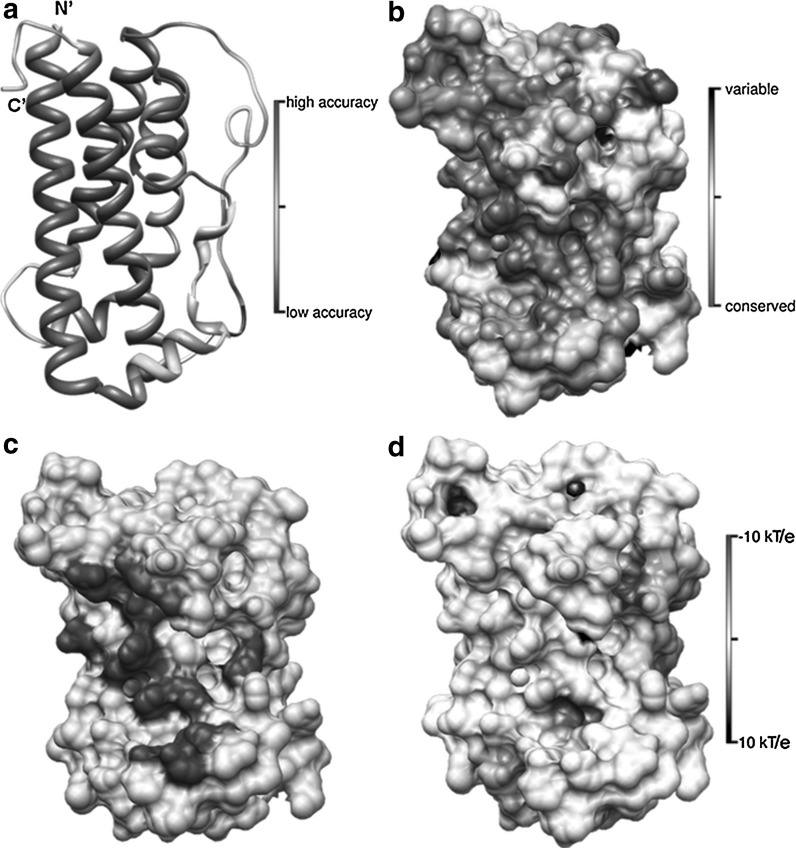
Analysis of the *T. tinca* structural GH model. **a** Results of the model quality evaluated by MetaMQAPII. **b** Surface conservation score of the GH model achieved in ConSurf based on the multiple alignment of 85 amino acid sequences of fish GH. **c** Localization of the predicted GH binding I site (*red*) for GHR. **d** The model highlighted with *colours* according to the distribution of electrostatic potential on the GH surface. (Color figure online)

Analysis of the full-length sequence of the growth hormone of *T. tinca* revealed nearly one hundred piscine orthologs with an significant sequence similarity (with e value above 0.005). Based on the amino acid alignment, the tench was placed within the Cyprinidae family what correlated with results obtained based on DNA comparisons (Kocour and Kohlmann 2011). In the obtained set of protein sequences, any fish growth hormone structural homologue was found in the Protein Data Bank (Berman et al. 2000). However, the tench GH sequence also showed significant similarity to six human growth hormone sequences with described 3D structures, PDB ID: 1HWG, 1KF9, 1HWH, 1HHR, 1HGU and 1AXI. The secondary structure predictions by Psi-Pred revealed a characteristic α-α-α-α pattern (Fig. 5a) that is well known for human GH. This pattern consists of four helices arranged in an up-down bundle type of fold (CATH, 1.20) (Greene et al. 2007). The fold recognition performed on several servers suggested that the 1AXI, 1HUW, 1HGU and 1A22 human GH structures are the most useful templates (top positions in ranking) for homology modelling of the tench growth hormone protein.

### Homology modelling

According to the 3D-jury scores, the most suitable reference template PDB ID: 1AXI chain A with the score 135.50 (FFAS3, score—81.5) was chosen for the homology modelling. The sequence similarity between the full target sequence (with the signal peptide) and the template calculated on alignment from the fold recognition was 29 %. The alignment covered almost the full length of the target sequence, and only one longer gap occurred in the region predicted by Psi-Pred, as loop, starting with K143 to F174. The best model was evaluated by the ProQ method as “very good” with LG score 3.309. The global accuracy was predicted as high by MetaMQAPII with RMSD (root-mean-square deviation) to native structure (currently unknown) 3.4 Å and GDT-TS score 54.8 (Fig. 5a).

## Discussion

Cultivating tench in Poland has a long tradition; however, the slow growth is a serious problem, which makes farming of this fish unattractive. The present study aims at the characterization of the tench growth hormone gene sequence and its protein. The information obtained in this work will be used for nutrigenomic studies in future where the influence of nutrition ingredients on the expression level of the somatotropic axis genes will be assessed. The structure of the tench *gh* resembles corresponding genes described for other species in the Cyprinidae family and comprises of 5 exons and 4 introns (Rajesh and Majumdar 2007). All analysed sequences were identical in length and base composition. This result likely depends on the sampling method where all fish were collected form one population. Such a bias is to be avoided in further analyses. In most cases, the sequences correlated with sequences in the NCBI database, and differences were only seen as a mispairing or short indels in intron regions. Non-coding *gh* regions that might have been mistakenly considered as useless may play an important role in regulation of growth hormone gene expression, as described for *Sparus aurata* (Almuly et al. 2000). Therefore, appropriate attention should be devoted to asses any possible correlation between polymorphisms and growth performance (Kocour and Kohlmann 2011).

Since decades, the wild stocks of tench populations are shrinking, and different approaches have been applied to bring them again to appropriate levels. Many artificial breading techniques have been developed (Kucharczyk et al. 2007), different food compositions were prepared for fry (Wolnicki et al. 2006) or tench density experiments were conducted (Celada et al. 2007) to find the best solution how effectively breed and culture this fish.

**Fig. 6:**
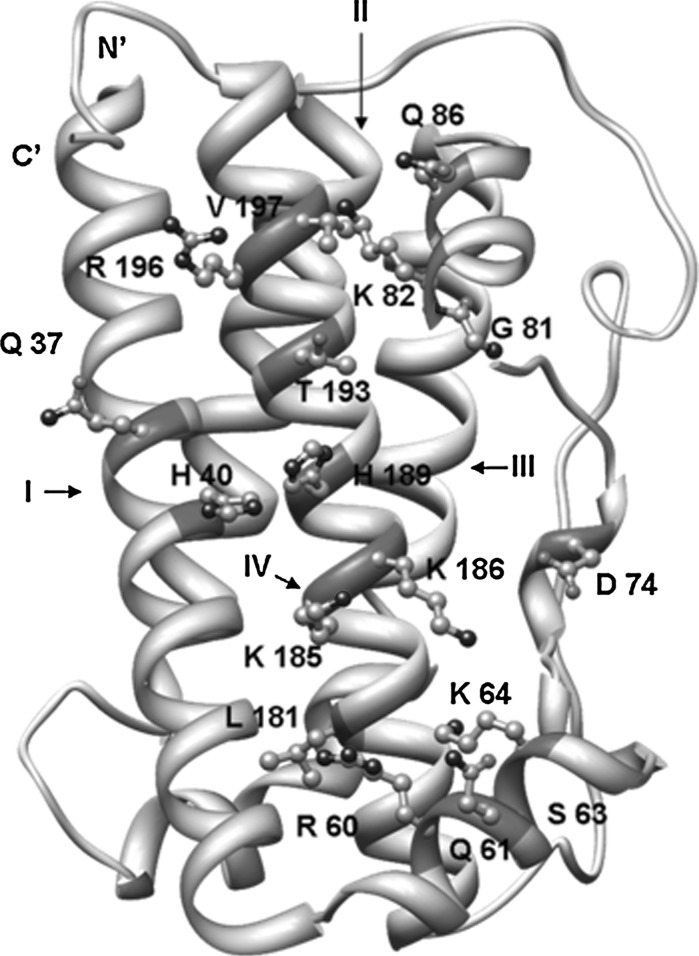
Predicted amino acid residue determinants of the binding site I for the *T. tinca* growth hormone

Therefore, the second aim of our work was to characterize and predict protein structure of tench growth hormone. GH protein binds to GHR (growth hormone receptor) by two regions I and II that are placed at the opposite sites of the analysed molecule (Wells et al. 1993; Walsh et al. 2003). The identification of binding sites (I, II) in the tench GH model was achieved by the superimposition of a known human growth hormone (PDB ID: 1A22) structure (RMSD Cα 0.56 Å) (Clackson et al. 1998). The recognized binding site I was placed in the central part of *T. tinca* GH (Fig. 5c). Structural alignments revealed that despite of the low overall sequence homology, the amino acid determinants binding site I of tench GH were similar to the human GH. Conservation mapping applied for the molecular surface of the GH model revealed that site I represents more conservative properties than the region located on the other side of analysed molecule (Fig. 5b). Most of the conserved residues were deployed in cluster at the C-terminus of the IV helix in the middle of the site I (Fig. 6). The predicted determinants in the adjacent helices I and III had distinctly higher variability.

The specificity of hormone–receptor binding greatly depends on the electrostatic properties of the molecules (Demchuk et al. 1994). Most determinants predicted in the present study were the polar amino acid residues that might form weak hydrogen bonds and the more stable salt bridges with oppositely charged residues of the receptor (Fig. 6). The presented results clearly indicate that the predicted binding site (positively charged) area is surrounded by a negatively charged rim (Fig. 5d). This is a defining characteristic of the transient interactions between proteins which are, inter alia, impacts between the hormone and the receptor (Nooren and Thornton 2003). The middle region of the IV helix is H 189, which occurs in all aligned GH fish amino acid sequences used in this study. The corresponding position of the human growth hormone is a conserved aspartic acid (D 171, PDB ID: 1A22). This residue was described as the major amino acid responsible for the binding specify of hGH by the formation salt bridge with R 43 of the hGHR (Behncken et al. 1997). Substitution of this residue in the hGH by a histidine decreases its affinity to the receptor hGHr that was described as species specifying factor (Souza et al. 1995). An alignment of the known fish protein GHr sequences (results not presented) and the human GHr showed that in complementary position to hGHR arginine 43 in the fish sequence, a glutamine that is surrounded by two glutamic acids can be found. This constellation can form a strongly negatively charged cluster on the receptor surface. The major affinity specifying factor between the tench GH and GHR can depends on the interaction between histidine 189 and glutamic acid residues. Another predicted GH-GHR binding determinates are well retained in all compared in this study fish GH protein sequences. However, this suggests that the problem of the slow growth tench in aquacultures compare to another species from Cyprinidae is not directly related with hormone structure. Probably, process may impact on another point in somatotropic axis.
